# The Effects of the Rearing System, Genotype at the *RYR1* Locus and Sex of Fattening Pigs on Selected Biochemical Blood Parameters and Concentrations of Cortisol and β-Endorphin

**DOI:** 10.3390/ani16193055

**Published:** 2026-09-28

**Authors:** Ewa Skrzypczak, Monika Greguła-Kania, Kinga Kropiwiec-Domańska, Karolina Szulc, Piotr Luciński, Bożena Dąbrowska, Maciej Feddek, Łukasz Szwarc, Łukasz Izbaner, Marek Babicz, Maria Kliks

**Affiliations:** 1 Department of Animal Breeding and Product Quality Assessment, Poznań University of Life Sciences, Szydłowska 50, 60-635 Poznań, Poland; 2 Department of Animal Breeding and Agricultural Consulting, University of Life Sciences in Lublin, Akademicka 13, 20-950 Lublin, Poland; marek.babicz@up.edu.pl; 3 Pstra Wybiegana, Nowy Dwór 75, 86-010 Koronowo, Poland; 4 Anteeo, Stary Jasiniec 71, 86-010 Koronowo, Polandizbaner@anteeo.com.pl (Ł.I.); 5 Faculty of Medicine, Poznań University of Medical Sciences—UMP, 60-812 Poznań, Poland

**Keywords:** pork, animal welfare, rearing system, local pig breeds, *RYR1* locus

## Abstract

There has been a growing interest in alternative rearing systems, including free-range rearing. This is particularly important in the case of indigenous pig breeds, which are prized for the high quality of their meat and traditional products, and whose importance in the production of high-quality food is steadily increasing. The aim of this study was to assess the effect of the rearing system (pen/free-range), genotype at the *RYR1* locus and sex of fattening pigs on cortisol and β-endorphin concentrations, as well as the parameters of protein, lipid and electrolyte metabolism, and indicators of liver and kidney function. This study was carried out on 120 Złotnicka Spotted breed fattening pigs raised under two rearing systems: pen housing (*n* = 60) and free-range (*n* = 60). The rearing system had the greatest impact on the biochemical indicators analysed in relation to the assessment of pig welfare. In free-range pigs, the observed changes included increases in total protein (8.04%), albumin (4.7%), Ca (3.4%), total cholesterol (11.7%), and triglycerides (107%), but lower urea (44.4%) and glucose (10.7%) concentrations compared with pen-reared pigs. The effect of the rearing system on the selected biochemical parameters was dependent on the *RYR1* genotype, sex or body weight of the animals, while for cortisol and β-endorphin, no significant interactive effects were demonstrated. By contrast, the effect of sex on the biochemical parameters analysed was minor.

## Simple Summary

The Złotnicka Spotted is an indigenous pig breed, which is prized for the high quality of its meat and traditional products. Rearing Złotnicka Spotted pigs in a free-range system does not adversely affect their health nor lead to chronic physiological stress. The changes observed in the selected biochemical parameters indicate that the animals have adapted metabolically to environmental conditions associated with greater exposure to external stimuli. At the same time, the absence of any signs of tissue damage confirms this breed’s good ability to adapt to free-range rearing. The results obtained indicate that this system may offer a valuable alternative to pen rearing, ensuring a high standard of animal welfare and aligning with the principles of sustainable, ethical, and consumer-friendly pigmeat production.

## 1. Introduction

In intensive pig farming systems, the animals are kept indoors throughout the entire rearing period, which significantly restricts their physical activity and their ability to engage in natural behaviours [1]. In recent years, however, there has been a growing interest in alternative rearing systems, including free-range rearing, which provides animals with more space, the opportunity to explore their environment, and greater freedom for physical activity [2,3]. This is particularly important in the case of indigenous pig breeds, such as the Złotnicka Spotted, which is prized for the high quality of its meat and traditional products, and whose importance in the production of high-quality food is steadily increasing.

At the same time, it should be emphasised that, despite its numerous benefits for animal welfare, the free-range system may also present additional environmental challenges. Animals reared in a free-range system are exposed to changing weather conditions, temperature fluctuations, precipitation, solar radiation and greater contact with biological factors, such as parasites or microorganisms present in the environment [4,5]. These factors may constitute a source of environmental stress and modify the physiological, metabolic and immunological response of the body [6,7]. On the other hand, greater physical activity and the opportunity to engage in natural behaviours may help to reduce the chronic stress associated with limited space and the monotonous environment characteristic of rearing in pens [3,8].

Previous studies on the effect of housing system on the biochemical profile of pigs often include commercial breeds, such as the Duroc [9], while data on indigenous breeds remain limited. One of the few examples is the study by Đidara et al. [10], which showed differences in biochemical and haematological parameters in pigs of different genotypes kept in a free-range system. In particular, there is a lack of studies that comprehensively assess the metabolic adaptation of this native breed to free-range conditions.

Biochemical blood parameters are a fundamental tool for assessing the physiological condition of humans and animals. They reflect the body’s metabolic state and the functioning of internal organs (including the liver and kidneys), as well as protein, lipid, and electrolyte balance [11]. Furthermore, they represent the body’s response to endogenous and exogenous factors, including stressors and physical activity [12]. Analysis of these parameters enables an assessment of the animal’s health and physiological function, and thus serves as an objective biomarker used in the assessment of pig welfare [13,14].

An important complement to this assessment is the measurement of hormone concentrations associated with the stress response, particularly cortisol [15,16]. Cortisol is the main glucocorticoid synthesised as a result of the activation of the hypothalamic–pituitary–adrenal (HPA) axis in response to a stressor. It plays a significant role in regulating metabolic processes, the immune response, and the body’s adaptive mechanisms; it is therefore regarded as an important biomarker of stress that indirectly indicates the level of well-being [17,18]. It should be mentioned that cortisol is not merely a stress marker; it is a hormone with multidirectional effects, including the regulation of energy homeostasis. Higher basal cortisol concentrations observed in some indigenous pig breeds may be associated with breed-specific differences in HPA-axis regulation, energy metabolism and adaptation to environmental conditions, rather than necessarily indicating a higher level of chronic stress [19,20]. An additional neuroendocrine biomarker is β-endorphin, which belongs to the group of endogenous opioid peptides and plays a significant neuromodulatory role in pain perception and the regulation of the body’s response to stress, both at the level of the HPA axis, as well as the autonomic nervous system [21,22]. According to Drolet G. et al. [23], opioid peptides reduce the negative effects of stress on the body by alleviating a range of physiological responses.

The role of both cortisol and β-endorphin in the regulation of metabolic processes means that their levels may correlate with the selected biochemical blood parameters that reflect the body’s metabolic and health status, as well as its level of homeostasis [24,25]. Research to date shows that the physiological response in animals is determined both by endogenous factors, such as sex and age [26,27,28], and genotype within the *RYR1* gene [29], and by exogenous factors, including, among others, environmental conditions and husbandry practices [13]. These factors influence the activity of the hypothalamic–pituitary–adrenal axis and, consequently, the levels of stress hormones and the selected biochemical blood parameters. In previous decades, β-endorphin was extensively studied in pigs as a stress-related biomarker; nowadays, its use in welfare research has become rare [30]. Most recent studies evaluating rearing systems focus primarily on cortisol, behavioural indicators, and markers of the immune system. Consequently, the simultaneous assessment of cortisol and β-endorphin levels in this study provides additional information on the neuroendocrine response in pigs.

Furthermore, most of the available studies do, however, focus on high-yielding pig breeds. There is still a lack of comprehensive research on conservation breeds, including the Złotnicka Spotted breed of pig, which is characterised by a slower growth rate, a different metabolism, and greater resistance to adverse environmental conditions [31,32]. Thanks to the lower intensity of selective breeding, this breed may serve as an animal model characterised by more stable physiological homeostasis under extensive rearing conditions, which is significant for research into animal welfare and stress biomarkers.

Furthermore, the available literature rarely includes a simultaneous assessment of a wide range of biochemical blood markers. This research gap justifies the conduct of this study.

The aim of this study was to assess the effect of the rearing system (pen/free-range), genotype at the *RYR1*, and sex of Złotnicka Spotted breed pigs on the concentration of hormones associated with stress and pain responses, as well as the parameters of protein, lipid, and electrolyte metabolism, and indicators of liver and kidney function.

## 2. Materials and Methods

### 2.1. Animals and the Experimental Setup

The experiment was carried out at a breeding farm rearing Złotnicka Spotted pigs. The study sample comprised 120 fattening pigs reared in two systems: a pen system (*n* = 60) and a free-range system (*n* = 60). The experiment was carried out between March and November. In addition to being categorised by rearing system, the animals were also classified according to their genotype at the *RYR1* locus (NN, Nn and nn) and by sex (gilts and barrows, in a 1:1 ratio).

Blood samples were collected during the final fattening period, after the animals reached an average body weight of approximately 98 kg. Animal body weight was determined individually using a portable single-chamber Meier–Brakenberg platform scale equipped with an automatic tare function. This point was chosen to assess the animals’ physiological condition following long-term exposure to the analysed housing conditions. Blood samples were collected on the same day, during the morning hours (6:00–7:00 AM), simultaneously by two veterinarians, following a uniform protocol. Due to the high sensitivity of the cortisol concentration to manipulation and animal restraint, the procedure was organised to minimise the time from contact with the animal to blood collection. Additional activities performed on individual animals, which could have prolonged the procedure and posed an additional stress factor, were also limited. Blood was collected from the marginal ear vein.

Serum biochemical parameter analyses were performed at external commercial veterinary laboratories, in accordance with their validated diagnostic procedures. Detailed information regarding the analyser models, reagent manufacturers, and calibration procedures was provided internally by the laboratories and was not made available to the client.

The activities of alkaline phosphatase (ALP), alanine aminotransferase (ALAT), aspartate aminotransferase (AST), α-amylase (AMY), and γ-glutamyl transpeptidase (GGTP) were determined, as well as the concentrations of total protein, albumin, creatinine, urea, glucose, calcium (Ca), magnesium (Mg), sodium (Na), chloride, inorganic phosphate, total cholesterol, HDL and LDL cholesterol, and triglycerides. Non-HDL cholesterol was calculated as the difference between the total and HDL cholesterol concentrations; the HDL/CHOL ratio was also determined. The cortisol concentration was determined by enzyme-linked immunosorbent assay (ELISA) using 96-well microtiter plates. A standard curve was prepared using a blank and a series of standards with known cortisol concentrations. The cortisol concentration was determined from the obtained standard curve. The serum β-endorphin concentration was determined by ELISA using the Pig βEP (Beta-Endorphin) ELISA Kit (ELK Biotechnology Co. Ltd., Denver, CO, USA). Absorbance was measured at 450 nm using a MINDRAY MR96A spectrophotometer (Mindray Bio-Medical Electronics Co., Ltd., Shenzhen, China). The method had a sensitivity of 4.79 pg/mL, a detection range of 15.6–1000 pg/mL, and an intra-assay precision of <8%.

A mutation at the *RYR1* gene locus was assessed using the PCR-RFLP method, employing a hair shaft with its root collected from the nape of the neck, in accordance with the method described by Fujii et al. in 1991 [33].

### 2.2. Housing and Feeding Requirements

The animals came from the farm where the research was conducted. The farm produces native breeds of pigs in a closed cycle, is subject to performance evaluation, and herd books are maintained for the Złotnicka Spotted pigs. Animals were included in the experiment at a body weight of approximately 25 kg.

Under the pen system, 60 piglets and fattening pigs were kept in two pens, each measuring 4 × 8 m. In the free-range system, 60 animals were kept in a single fenced enclosure of approximately 5000 m^2^, with access to natural substrate. The enclosure allowed the animals to express their natural behaviours and provided them with constant access to water, feed and shelter.

The housing conditions and animal density in both systems were in line with applicable requirements for pig welfare and biosecurity. Individual animals served as observation units in the analyses of biochemical and hormonal parameters.

In both systems, single-phase ad libitum feeding was used. The feed mixture complied with the Programme for the Conservation of the Genetic Resources of the Złotnicka Spotted breed of pig and consisted of 35% barley, 35% triticale, 20% wheat and 10% lupin. The total protein content was 123.9 g/kg, and the metabolisable energy content was 12.53 MJ/kg. In addition, the animals were given whey and steamed potatoes.

In the pen system, the microclimate conditions met current recommendations, with temperatures ranging from 16 to 20 °C and relative humidity ranging from 65 to 75%. In the free-range system, the animals were exposed to natural weather conditions, with temperatures ranging from 5 to 25 °C and relative humidity ranging from 60 to 90%. There was no systematic recording of precipitation or sunshine.

### 2.3. Statistical Analysis

The collected data were analysed using SAS 9.4 statistical software [34]. Basic descriptive statistics, including the arithmetic mean, minimum and maximum values, standard deviation, coefficient of variation, and number of observations, were calculated using the MEANS and UNIVARIATE procedures.

The effects of the rearing system, genotype at the *RYR1* locus, and sex on the analysed biochemical blood parameters and on the cortisol and β-endorphin concentrations were assessed using a general linear model (GLM), with body weight on the day of blood sampling included as a covariate. Individual animals were treated as observational units for the analyses of biochemical and hormonal parameters. However, because all free-range pigs were maintained in a single enclosure, there was no independent replication at the level of the “rearing system” factor.

For the main dependent variables, i.e., cortisol, β-endorphin, ALAT, ASPAT, total protein, and urea, the model was extended to include interactions between the rearing system and *RYR1* genotype, the rearing system and sex, and the rearing system and body weight:

Y = μ + S + G + P + βMC + S × G + S × P + S × MC + e

where,

Y—the value of the analysed trait;
μ—the overall mean;
S—the fixed effect of rearing system;
G—the fixed effect of genotype at the *RYR1* locus;
P—the fixed effect of sex;
βMC—regression term describing the linear effect of body weight, where β is the regression coefficient, and MC is the animal’s body weight on the day of blood sampling, included as a covariate;
S × G—interaction between rearing system and *RYR1* genotype;
S × P—interaction between rearing system and sex;
S × MC—interaction between rearing system and body weight;
e—the random error.

Because no animals with the nn genotype were present in the pen system, the rearing system × *RYR1* genotype interaction was evaluated for the NN and Nn genotypes only. Tukey’s test was used for multiple comparisons of means. Statistical significance was accepted at *p* ≤ 0.05. Significant interactions were presented graphically. No additional global correction for multiple hypothesis testing, such as the Bonferroni, Holm–Bonferroni, or false discovery rate (FDR) procedure, was applied.

The assumptions of the statistical models were assessed based on residual diagnostics. The normality of residuals was evaluated using the Shapiro–Wilk test, whereas the homogeneity of variances was assessed using Levene’s test. For some of the analysed parameters, deviations from these assumptions were observed; therefore, the corresponding results were interpreted with caution.

### 2.4. Approval from the Ethics Committee

This research was carried out under production conditions as part of the breeding programme for the Złotnicka Spotted pig breed. The collection of biological material was carried out in accordance with § 3 (7 of the Regulations on the Rights and Obligations of the Poznań University of Life Sciences), which maintains herd books for the Złotnicka Spotted pig breed, and of breeders participating in the Złotnicka Spotted pig breed breeding programme. Biological material was collected as part of ongoing population monitoring, which included, among other things, genetic characterisation of the animals at the *RYR1* locus. The material obtained during these procedures was simultaneously used for the analyses presented in this paper, without the need for additional blood sampling for this study. All procedures were carried out by a veterinary surgeon in accordance with the applicable animal welfare guidelines. This study did not involve any experimental procedures within the meaning of the applicable regulations; therefore, it did not require approval from the Local Ethics Committee for Animal Experimentation.

## 3. Results and Discussion

### 3.1. The Effect of the Pen and Free-Range Systems on the Biochemical Blood Parameters in Pigs

A comparison of the selected biochemical parameters in pig blood revealed significant differences depending on the rearing system (Table 1).

Animals reared in a free-range system exhibited a different profile of physiological indicators compared with those reared in pen conditions, which indicates the influence of the environment on the functioning of the organism.

With regard to liver enzymes, significantly higher levels of ALP and ASPAT were observed in free-range animals. This may be linked to greater muscular and mechanical stress on the bones, as well as to bone remodelling in young individuals [35]. Greater physical activity and exertion (walking, digging, and interactions) in this group, compared with the indoor group, may cause cell membrane permeability, leading to elevated levels of these enzymes in the blood. Findings from other researchers indicate that the ASPAT value for the animal rearing system is not statistically significant [8]. The activity of this enzyme depends to a greater extent on the intensity of physical activity and other environmental factors, such as exposure to changing weather conditions (temperature, humidity, wind) [4,7,38]. They may increase physiological stress, which in turn affects metabolism and may subsequently raise ASPAT levels. In contrast, higher mean ALAT levels were observed in the pen system. This may reflect a metabolic adjustment to the living conditions. Intensive rearing systems expose animals to a range of chronic stressors—such as restricted living space or limited opportunities to express natural behaviours—which may affect metabolic homeostasis. It has been shown that these factors modify liver metabolism and may contribute to a moderate increase in serum liver enzyme levels [16]. However, given the lower levels of ASPAT in these same animals, the elevated ALAT level should be interpreted with caution; it is unlikely to indicate clinically significant liver damage. It should be emphasised that, although statistically significant differences were observed between the two groups in terms of certain biochemical serum parameters, the absolute values of all the measured indicators fell within the accepted physiological reference ranges for healthy pigs, with the exception of ALAT, which was slightly elevated in pigs in pen conditions [35,36,37].

The levels of total protein and albumin in the blood reflect the animal’s nutritional status, the liver’s ability to synthesise proteins, and the animal’s overall utilisation of amino acids. The parameters of energy and protein metabolism also differed between the groups. An increase in feed efficiency or protein utilisation usually results in higher total protein levels in the blood. Animals from the free-range system had some increase in the total protein and albumin levels (by only 8.0% and 5.2%, respectively), but urea levels decreased significantly (by 44.4%), compared with the pen-reared group. This may indicate a more efficient use of amino acids for the synthesis of body proteins rather than their catabolism. Urea is the end product of amino acid catabolism. The lower urea concentration observed in free-range pigs may be consistent with differences in protein and amino acid metabolism. However, because feed intake, nitrogen balance, and amino acid utilization were not directly assessed, the observed differences cannot be conclusively attributed to more efficient amino acid utilization or reduced protein catabolism [39]. Animals reared in a free-range system are exposed to greater variability in environmental conditions and exhibit greater spontaneous physical activity. These factors promote metabolic adjustments, which involve the more efficient utilisation of energy substrates and better utilisation of amino acids for the synthesis of body proteins. Consequently, a smaller quantity of amino acids undergoes deamination and is converted into urea, which may explain the lower concentration of urea in the serum of free-range pigs. Despite potentially higher levels of physical activity in the outdoor environment and the greater energy expenditure required for thermoregulation, individuals reared outdoors maintained an increased protein metabolism [9]. However, the lack of comprehensive monitoring of environmental conditions, particularly precipitation and sunlight, is a limitation of this study. Therefore, the potential influence of atmospheric conditions, thermoregulation, and sunlight exposure on the observed changes should be interpreted with caution. Albumin, the main protein synthesised by hepatocytes, reflects the liver’s synthetic capacity and acts as a transport protein for many endogenous compounds, including free fatty acids, bilirubin, and hormones [40]. When combined with higher levels of total cholesterol (by 11.7%) and triglycerides (by 107%), this may suggest a metabolic adjustment to greater physical activity and increased energy requirements in free-range animals.

Higher serum creatinine concentrations were observed in the free-range pigs (although the difference between the groups was not statistically significant), and this level was above normal in the pigs under free-range conditions. This may reflect their higher level of physical activity and faster muscle metabolism, rather than impaired kidney function. Similar observations were made by Hale et al. 1986 [41], who demonstrated significantly higher plasma creatinine concentrations in pigs exercised on a treadmill compared with the non-exercising control group.

Serum glucose and triglyceride levels are the main indicators of energy balance and lipid metabolism. Lipid metabolism is one of the most sensitive components of the stress response [13]. Free-range rearing requires a constant and higher energy intake, as it typically increases physical activity and thermoregulation, whilst the increased energy expenditure promotes the mobilisation of fat and liver glycogen reserves [5]. Higher levels of total cholesterol, triglycerides and non-HDL cholesterol may reflect differences in lipid metabolism associated with the housing system. Physical activity affects lipid metabolism, leading to the mobilisation of lipids from adipose tissue and an increase in the transport of lipids in the blood, as demonstrated in pigs subjected to increased physical activity [8,9]. Increased physical activity and altered energy expenditure represent possible explanations; however, physical activity and energy balance were not directly measured in the present study. At the same time, lower glucose (by 10.7%) and urea concentrations (44.4%) may indicate more efficient utilisation of energy substrates and a reduction in protein catabolism.

Conversely, the significantly higher blood urea levels observed in the animals reared in a pen system may indicate that rearing conditions affect the animals’ activity levels and can alter protein metabolism and the efficiency of nitrogen utilisation. In the pen system, energy requirements are lower (due to reduced movement and thermoregulation), so a larger proportion of amino acids undergo deamination, which ultimately leads to an increase in urea production [39]. The higher total protein concentration (by 8%) and lower urea concentration (by 44.4%) observed in the free-range pigs may indicate a more efficient utilisation of amino acids for the synthesis of body proteins. This interpretation is supported by research by Hayes et al. [9], who found higher concentrations of total protein in pigs reared in an outdoor system. In a study by Berghaus et al. [39], a negative correlation was demonstrated between blood urea nitrogen (BUN) levels and nitrogen utilisation efficiency, suggesting that lower urea levels reflect more efficient utilisation of amino acids for protein synthesis.

Calcium, sodium, and magnesium perform key physiological functions in pigs, contributing, respectively, to bone formation and the regulation of muscle contraction (Ca), maintaining water and electrolyte balance and nerve conduction (Na), and acting as enzyme co-factors and regulators of neuromuscular excitability (Mg) [42].

In terms of mineral metabolism, the free-range pigs had higher calcium (by 3.3%) and sodium (by 2.3%) levels, whilst their magnesium (by 7.4%) and inorganic phosphate (by 7.5%) concentrations were lower. The higher calcium concentration observed in the free-range pigs may be associated with differences in mineral metabolism. Greater sunlight exposure represents an explanation, as ultraviolet B exposure can contribute to vitamin D3 synthesis in the skin, which in turn increases calcium absorption in the gut and bone mineralisation and, consequently, influences calcium homeostasis; however, vitamin D status was not assessed in the present study [43]. Conversely, the higher concentrations of inorganic phosphorus and magnesium in pigs reared in a pen-based system may reflect differences in mineral metabolism, resulting from varying levels of physical activity. Both phosphorus and magnesium play a key role in energy metabolism, as they are essential components of adenosine triphosphate (ATP) and cofactors for numerous enzymes. The higher level of physical activity among the free-range pigs may have led to more intensive utilisation of these components by the tissues, which may have contributed to their slightly lower serum concentrations. All parameters relating to mineral metabolism—Ca, Na, inorganic phosphates, and chlorides—were within the physiological range, with the exception of magnesium, which was slightly deficient; these differences do not disrupt electrolyte balance, indicating that the subjects in both systems maintained osmotic balance and hydration status, and that the differences may result from natural adaptive changes to increased physical activity [35,38].

It is also worth paying attention to neuroendocrine stress markers. Cortisol is the most commonly used marker of activation of the hypothalamic–pituitary–adrenal (HPA) axis. Cortisol is released into the bloodstream by the adrenal glands; this process is triggered by adrenocorticotropic hormone (ACTH), the secretion of which is stimulated by the corticotropin-releasing factor. This sequence of interactions is known as the hypothalamic–pituitary–adrenal (HPA) axis. β-endorphin, on the other hand, is synthesised mainly in the anterior lobe of the pituitary gland and in the hypothalamus, and is released into the blood simultaneously with the adrenocorticotropic hormone (ACTH) during activation of the hypothalamic–pituitary–adrenal (HPA) axis [17]. The mean serum cortisol concentration was higher in pigs kept in the free-range system (183 nmol/L) than in the pigs housed indoors (149 nmol/L), whereas β-endorphin concentrations were very similar between the groups (954 vs. 940 pg/mL). However, neither of these differences was statistically significant. The considerable inter-individual variability in cortisol concentrations may reflect differences in individual HPA-axis responsiveness to environmental and handling-related stimuli. Although blood samples were collected during the morning hours, the exact time of sampling and duration of restraint were not incorporated into the statistical model. Therefore, a potential contribution of circadian variation and acute handling-related stress to the observed inter-individual variability cannot be completely excluded.

In this experiment, the absence of statistically significant differences in cortisol and β-endorphin concentrations between the housing systems suggests that the different housing conditions did not result in a statistically detectable difference in the measured endocrine stress-related responses. Given the concurrent differences in lipid metabolism (total cholesterol, triglycerides, and non-HDL cholesterol), this may reflect metabolic adjustments that may be associated with differences in physical activity and energy expenditure between the housing systems. The absence of correlation analysis between cortisol and metabolic parameters limits the extent to which a direct relationship between the stress response and the observed metabolic adjustments can be inferred.

An important limitation of this study was the lack of independent replication at the level of the “rearing system” factor, particularly because all of the free-range pigs were maintained in a single enclosure. Therefore, individual animals cannot be considered independent replicates of the rearing-system treatment. Consequently, the observed differences between the groups should be interpreted cautiously as associations observed under the conditions of the present study, without attributing them directly to causal effects of the rearing system or making overly broad generalisations.

In studies on Pietrain and Duroc breeds following stress (ear tagging), the increase in cortisol was lower in outdoor pigs; the post-stress plasma cortisol levels did not differ significantly between the groups, despite marked behavioural differences [3]. The lack of a clear-cut interpretation of cortisol is consistent with the findings of other authors, as this hormone exhibits considerable variability and should be interpreted in conjunction with metabolic markers, i.e., behaviour, production parameters and immunity indicators [1,13].

The cortisol levels observed in this study are similar to those reported by Rosochacki et al. [44] as a result of herding pigs or during the initial phase of their brief immobilisation. These values correspond to the range observed in pigs subjected to moderate arousal or short-term stress, but are significantly lower than those recorded during severe immobilisation, intense stimulation of the HPA axis, or following ACTH stimulation.

In a study by Zanella et al. [30], sows kept in groups were compared with sows kept in individual pens. The authors found no correlation between the type of rearing system and β-endorphin concentrations; however, the animals’ social status had a greater impact than the rearing system itself. Numerous studies indicate that both cortisol and β-endorphin are components of the stress response, but neither should be interpreted in isolation from other indicators [17].

### 3.2. The Effect of the RYR1 Genotype on the Biochemical Blood Parameters in Pigs

The statistical analysis showed that the *RYR1* genotype had a significant effect on certain biochemical and enzymatic parameters in the blood of pigs (Table 2).

The most marked differences were observed in liver enzyme activity and the selected protein parameters.

A *RYR1* mutation causes the uncontrolled release of Ca^2+^ from the sarcoplasmic reticulum of muscles, leading to prolonged muscle contractions, increased ATP consumption, accelerated glycolysis, and damage to the cell membranes of muscle fibres [45].

In the pigs with the nn genotype (homozygous recessive), which is associated with the presence of a mutation in the *RYR1* gene (linked to stress susceptibility and PSS syndrome), significantly higher levels of ALAT, ASPAT and AMY were observed compared with NN and Nn individuals (*p* ≤ 0.01). AST is an enzyme whose increase best reflects muscle damage in pigs with PSS (Porcine Stress Syndrome). In the study by Popovski et al. 2016 [29], the effect of the *RYR1* genotype on ASPAT levels was reported for the first time, and these levels were three times higher than the upper limit of the reference range in pigs with the nn genotype. Our research confirms this, as the pigs with the nn genotype in the *RYR1* gene have ASPAT levels that are twice the upper limit of the reference range [37]. In situations involving extremely high muscle activity, AST and ALT are released into the bloodstream as a result of a breach in the integrity of the cell membrane and changes in the permeability of muscle cells. Furthermore, individuals who are homozygous for the nn genotype at the *RYR1* locus are characterised by a higher lean meat content and, consequently, a greater potential for an increase in the activity of stress-related enzymes in the blood, as skeletal muscle is the main source of AST and ALT [45]. Higher AMY activity in homozygous recessive individuals may indicate stronger activation of the sympathetic nervous system in pigs susceptible to stress [29], however, given the lack of studies directly analysing the relationship between the *RYR1* genotype and the activity of this enzyme, this interpretation requires further analysis.

Conversely, higher concentrations of albumin—a negative acute-phase protein—in homozygous dominant NN individuals may indicate a more stable metabolic state and lower activation of the acute-phase response.

These results are consistent with observations made in pigs during transport, as animals carrying the n allele had lower albumin concentrations [45].

The other parameters analysed, including glucose, lipid profile (total cholesterol, HDL, and LDL), electrolytes, urea, and total protein, did not show any statistically significant differences (*p* > 0.05) among the genotype groups. This means that the *RYR1* genotype did not have a significant effect on most of the metabolic parameters in the study population. No significant effect of the *RYR1* genotype on cortisol concentration was observed (*p* = 0.517), indicating that the observed numerical differences between NN, Nn, and nn animals should not be interpreted as a genotype-dependent response.

### 3.3. The Effect of Sex on the Biochemical and Morphological Blood Parameters in Pigs

A comparative analysis by sex (Table 3) showed that most of the biochemical and morphological blood parameters assessed did not differ statistically significantly (*p* > 0.05) between the gilts and barrows, which indicates the overall homeostatic stability of these parameters regardless of the animals’ sex.

Of all the indicators examined, two parameters showed statistically significant differences between the gender groups. These were: gamma-glutamyltransferase (GGTP) activity and serum albumin concentration.

The gilts exhibited significantly higher GGTP activity compared with barrows, which may suggest greater involvement of the detoxification pathway in liver cells in females under the conditions studied. At the same time, the gilts showed higher albumin concentrations, which may indicate their better protein status or physiological differences in plasma metabolism between the sexes.

There are currently no findings regarding the effect of sex on albumin levels in production pigs. However, studies on Göttingen minipigs have shown that 48-week-old female minipigs had higher albumin concentrations than 48-week-old males [46]. The other parameters, including enzymatic parameters (ALT, AST, and alpha-amylase), indicators of protein, carbohydrate, and lipid metabolism, electrolytes, and hormones, showed no significant differences between the females and males.

### 3.4. Analysis of Interaction Effects

Additional analysis of the main dependent variables revealed significant interactions between the housing system and the *RYR1* genotype, sex, or body weight for selected biochemical parameters.

**Figure 1:**
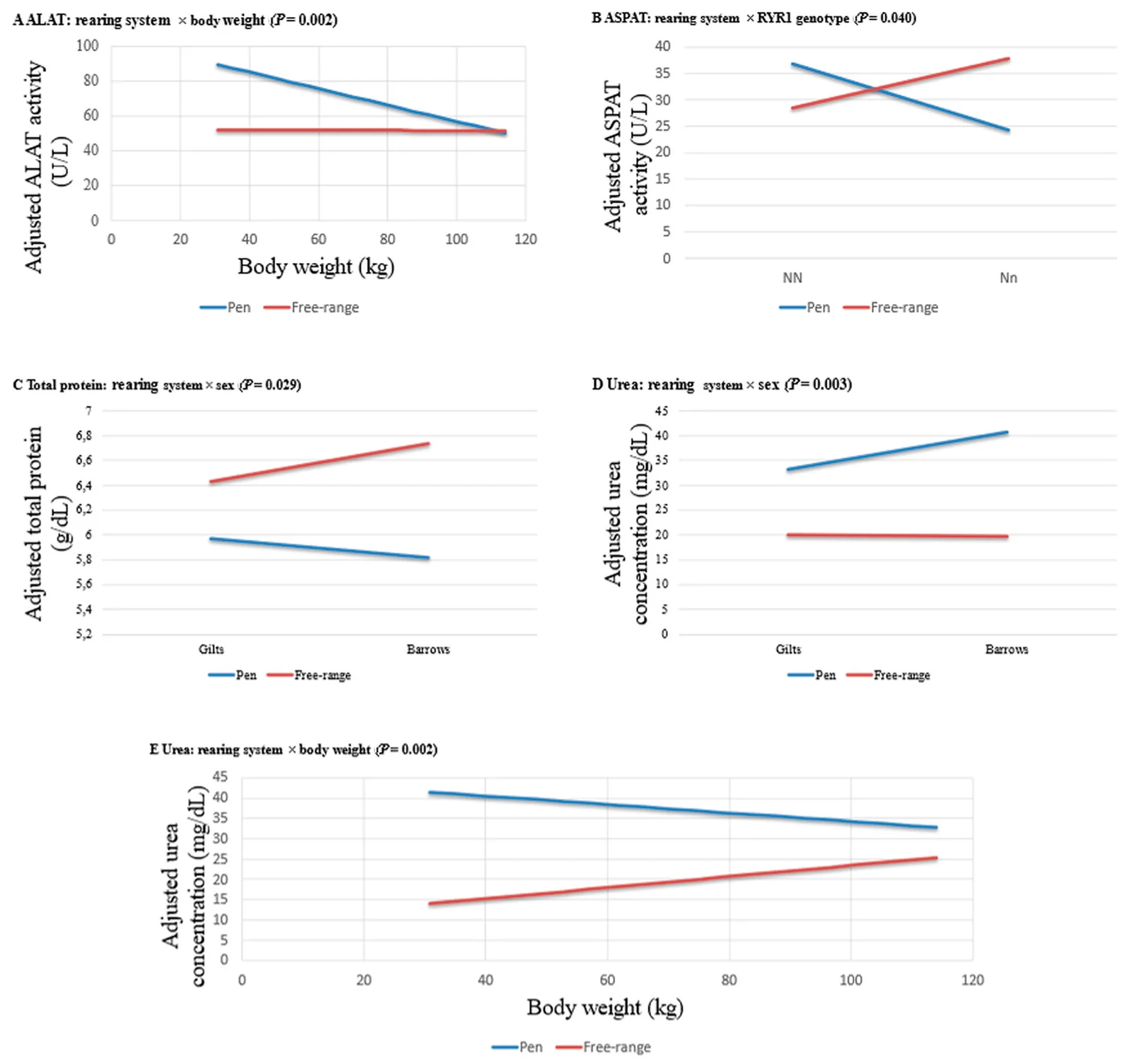
Significant interaction effects between rearing system and selected factors for biochemical blood parameters in Złotnicka Spotted fattening pigs: (**A**) rearing system × body weight for ALAT activity (*p* = 0.002); (**B**) rearing system × *RYR1* genotype (NN and Nn) for ASPAT activity (*p* = 0.040); (**C**) rearing system × sex for total protein concentration (*p* = 0.029); (**D**) rearing system × sex for urea concentration (*p* = 0.003); and (**E**) rearing system × body weight for urea concentration (*p* = 0.002).

A significant housing system × body weight interaction was found for ALAT activity (*p* = 0.002; Figure 1A). In the pen system, ALAT activity decreased with increasing body weight, whereas in the free-range system, this relationship was significantly weaker. The housing system may, therefore, modulate the relationship between body weight and ALT activity, which is consistent with the growth-stage-dependent changes in ALT reported by Đidara et al. [10].

For AST activity, a significant housing system × *RYR1* genotype interaction was found (*p* = 0.040; Figure 1B). In the pen system, AST activity was higher in NN than Nn individuals, whereas in the free-range system, the opposite relationship was observed. However, this result should be interpreted with caution due to the lack of individuals with the nn genotype in the pen system. For the total protein concentration, a significant housing system × sex interaction was observed (*p* = 0.029; Figure 1C). In the free-range system, the total protein concentration was higher in the barrows than in the gilts, whereas in the pen system, the difference between sexes was smaller and in the opposite direction. These results indicate that the effect of housing system on the total protein concentration may differ between sexes, which is consistent with previous studies on the effect of free-range [9] and sex [28] on this parameter.

Significant housing system × sex (*p* = 0.003; Figure 1D) and housing system × body weight (*p* = 0.002; Figure 1E) interactions were found for the urea concentration. In the pen system, the urea concentration was higher in the barrows than in gilts, whereas in the free-range system, the sex differences were small. The relationship between body weight and the urea concentration was negative in the pen system and positive in the free-range system. These results indicate that the effect of the housing system on the urea concentration was modified by both the sex and body weight of the animals. This is consistent with previous observations indicating a relationship between the urea concentration and sex and growth stage of pigs [27] and housing conditions [6]. For the cortisol and β-endorphin concentrations, no significant interactions were found between the housing system and the analysed factors (*p* > 0.05). This indicates that the effect of the housing system on these neuroendocrine markers was not significantly modified by the analysed factors.

Due to the lack of an additional global correction for multiple hypothesis testing, the results with *p* values close to the assumed significance level (*p* ≤ 0.05) should be interpreted with caution.

The obtained results indicate that the effect of the housing system on the selected biochemical parameters was dependent on the *RYR1* genotype, sex, or body weight of the animals, whereas no significant interaction effects were observed for cortisol and β-endorphin.

## 4. Conclusions

Animals reared in a free-range system exhibited a different profile of physiological indicators compared with those reared in pen conditions, which indicates the influence of the environment on the functioning of the organism. This may reflect metabolic adjustment to the living conditions.

Significantly higher levels of ALP and ASPAT in free-range animals may be linked to greater physical activity and exertion (walking, digging, and interactions) in this group. Moreover, in this group, we observed an increase in feed efficiency and protein utilisation, and higher total protein and albumin levels in the blood.

Furthermore, Złotnicka Pstra pigs reared in a free-range system are exposed to greater variability in environmental conditions and exhibit greater spontaneous physical activity. These factors promote metabolic adjustment, which probably involves the more efficient utilisation of energy substrates and better utilisation of amino acids for the synthesis of body proteins. The lower urea concentration observed in the free-range pigs may be consistent with differences in protein and amino acid metabolism. However, because feed intake, nitrogen balance, and amino acid utilisation were not directly assessed, the observed differences cannot be conclusively attributed to more efficient amino acid utilisation or reduced protein catabolism. The higher levels of total cholesterol, triglycerides and non-HDL cholesterol may reflect differences in lipid metabolism associated with the housing system. Physical activity affects lipid metabolism, leading to the mobilisation of lipids from adipose tissue and an increase in the transport of lipids in the blood in the free-range system.

In this experiment, rearing the pigs in either a free-range or pen system had no significant effect on blood levels of cortisol and beta-endorphin. The absence of statistically significant differences in cortisol and β-endorphin concentrations between the housing systems suggests that the different housing conditions did not result in a statistically detectable difference in the measured endocrine stress-related responses. We emphasise that these findings should not be equated with a comprehensive assessment of animal welfare. To verify whether this system can serve as a valuable alternative to pen-based rearing, aligning with the principles of sustainable, ethical, and consumer-friendly pork production, a comprehensive welfare assessment is required. This assessment should integrate physiological, behavioural, health, and management-related indicators, ideally evaluated repeatedly throughout the entire rearing period.

With regard to the genotype at the *RYR1* locus, it was shown that animals with the nn genotype exhibited severe metabolic disorders, including significantly elevated levels of liver enzymes (ALAT and ASPAT) and alpha-amylase and reduced serum albumin levels, which may indicate their reduced adaptive capacity and an increased risk of homeostasis disorders. Although the results obtained for the nn genotype may indicate potentially relevant physiological differences, the small number of animals carrying this genotype (*n* = 4) does not allow definitive conclusions regarding its suitability for breeding. Further studies involving larger populations are required before any recommendations concerning the management or elimination of this genotype can be formulated. The effect of sex on the parameters analysed was minor and was limited to higher GGTP activity and higher albumin concentrations in gilts compared with barrows. The results obtained may serve as a basis for further development of breeding and management guidelines supporting the implementation of animal-friendly rearing systems which are in line with the expectations of consumers and principles of sustainable pig production.

## Figures and Tables

**Table 1:** The effect of the rearing system (pen-reared vs. free-range) on the biochemical blood parameters in fattening pigs.

Biochemical Blood Parameters	Rearing System						SE	*p*	Reference Interval
	Pen			Free-Range					
	*n*	$\bar{x}$	SD	*n*	$\bar{x}$	SD			
ALP (U/L)	60	116.32 ^A^	18.57	60	141.71 ^B^	39.67	4.89	0.006	118–395
ALAT (U/L)	60	66.16 ^B^	11.65	60	53.74 ^A^	11.24	1.74	<0.001	31–58
ASPAT (U/L)	60	28.16 ^A^	11.19	60	47.09 ^B^	5.32	6.00	0.001	32–84
AMY (U/L)	60	2647.74	427.72	60	2793.40	744.67	88.48	0.643	739–2882
GGTP (U/L)	60	32.74	11.29	60	36.29	11.17	1.53	0.474	10–60
Total protein (g/dL)	60	5.97 ^A^	0.22	60	6.45 ^B^	0.43	0.06	<0.001	5.4–8.9
Albumin (g/dL)	60	4.24 ^A^	0.43	60	4.46 ^B^	0.44	0.06	0.004	3.1–5.3
Creatinine (mg/dL)	60	1.34	0.14	60	5.27	2.31	2.53	0.258	1–2.7
Urea (mg/dL)	60	35.33 ^B^	5.90	60	19.63 ^A^	4.31	1.22	<0.001	10–30
Glucose (mg/dL)	60	89.53 ^B^	13.21	60	79.97 ^A^	6.72	1.42	0.005	85–150
Ca (mg/dL)	60	9.70 ^A^	0.37	60	10.02 ^B^	0.35	0.05	0.001	9–11
Mg (mg/dL)	60	2.43 ^B^	0.18	60	2.25 ^A^	0.16	0.03	0.001	2.7–3.7
Na (mmol/L)	60	140.81 ^A^	1.80	60	144.02 ^B^	2.00	0.33	<0.001	131–151
Chlorides (mmol/L)	60	102.76	2.86	60	102.77	2.52	0.36	0.832	28–153
Inorganic phosphates (mg/dL)	60	8.30 ^B^	0.46	60	7.68 ^A^	0.59	0.08	<0.001	4–8
Total cholesterol (mg/dL)	60	80.16 ^A^	8.05	60	89.54 ^B^	8.95	1.32	<0.001	28–153
Triglycerides (mg/dL)	60	19.68 ^A^	5.40	60	40.77 ^B^	9.53	1.78	<0.001	8–114
HDL cholesterol (mg/dL)	60	39.21	4.05	60	41.17	4.77	0.62	0.104	-
LDL cholesterol (mg/dL)	60	37.21	5.53	60	40.29	6.39	0.85	0.054	-
HDL/CHOL	60	48.84 ^B^	3.48	60	46.20 ^A^	4.21	0.56	0.039	-
Non-HDL cholesterol (mg/dL)	60	41.21 ^A^	5.64	60	48.37 ^B^	6.80	0.99	<0.001	-
Cortisol (nmol/L)	60	149.82	78.40	60	183.64	64.55	9.65	0.071	-
β-endorphins (pg/mL)	60	940.41	371.17	60	954.10	376.37	50.50	0.614	-

ALAT—alanine aminotransferase; ALP—alkaline phosphatase; ASPAT—aspartate aminotransferase; AMY—alpha-amylase; GGTP—gamma-glutamyl transpeptidase. Significance of the factor under analysis: *p* ≤ 0.01—highly significant; 0.01 < *p* ≤ 0.05—significant; *p* > 0.05—not significant. The means denoted by the different letters (A, B) differ statistically significantly (*p* ≤ 0.05). The reference interval was assessed according to [35,36,37].

**Table 2:** The effect of the genotype at the *RYR1* locus (NN, Nn, nn) on the biochemical blood parameters in pigs.

Biochemical Blood Parameters	*RYR1*									SE	*p*
	NN			Nn			nn				
	*n*	$\bar{x}$	SD	*n*	$\bar{x}$	SD	*n*	$\bar{x}$	SD		
ALP (U/L)	54	120.40	34.87	62	139.10	35.45	4	154.00	2.83	4.89	0.823
ALAT (U/L)	54	55.86 ^A^	10.15	62	57.77	12.61	4	87.00 ^B^	5.66	1.74	<0.001
ASPAT (U/L)	54	29.52 ^A^	12.76	62	34.87 ^A^	17.73	4	241.00 ^B^	79.20	6.00	<0.001
AMY (U/L)	54	2557.76 ^A^	553.49	62	2787.29 ^A^	635.30	4	3978.50 ^B^	581.95	88.48	0.008
GGTP (U/L)	54	33.62	11.07	62	35.58	11.70	4	41.50	0.71	1.53	0.846
Total protein (g/dL)	54	6.32	0.50	62	6.26	0.40	4	6.05	0.07	0.06	0.679
Albumin (g/dL)	54	4.47 ^B^	0.44	62	4.37 ^B^	0.43	4	3.69 ^A^	0.04	0.06	0.031
Creatinine (mg/dL)	54	7.83	2.98	62	1.38	0.12	4	1.24	0.04	2.53	0.793
Urea (mg/dL)	54	26.47	8.45	62	24.66	9.52	4	19.10	5.09	1.22	0.872
Glucose (mg/dL)	54	85.38	14.48	62	82.26	6.91	4	78.50	2.12	1.42	0.980
Ca (mg/dL)	54	9.93	0.36	62	9.93	0.40	4	9.40	0.00	0.05	0.052
Mg (mg/dL)	54	2.32	0.21	62	2.32	0.17	4	2.15	0.21	0.03	0.720
Na (mmol/L)	54	142.88	2.15	62	143.00	2.73	4	141.35	0.78	0.33	0.164
Chlorides (mmol/L)	54	103.04	2.71	62	102.60	2.63	4	102.55	2.62	0.36	0.852
Inorganic phosphates (mg/dL)	54	8.00	0.54	62	7.82	0.68	4	7.96	0.69	0.08	0.467
Total cholesterol (mg/dL)	54	88.00	10.71	62	85.32	9.10	4	82.00	8.49	1.32	0.134
Triglycerides (mg/dL)	54	30.76	14.13	62	34.77	12.67	4	38.50	4.95	1.78	0.552
HDL cholesterol (mg/dL)	54	41.10	4.95	62	40.23	4.43	4	38.00	4.24	0.62	0.492
LDL cholesterol (mg/dL)	54	40.95	6.08	62	38.19	6.31	4	36.50	3.54	0.85	0.178
HDL/CHOL	54	46.81	4.17	62	47.42	4.29	4	46.00	0.00	0.56	0.666
Non-HDL cholesterol (mg/dL)	54	47.05	7.80	62	45.16	7.05	4	44.00	4.43	0.99	0.165
Cortisol (nmol/L)	54	161.38	81.64	62	178.86	65.53	4	170.20	20.51	9.65	0.517
β-endorphins (pg/mL)	54	1047.78	440.92	62	897.78	315.65	4	713.30	154.29	50.50	0.289

ALAT—alanine aminotransferase; ALP—alkaline phosphatase; ASPAT—aspartate aminotransferase; AMY—alpha-amylase; GGTP—gamma-glutamyl transpeptidase. Significance of the factor under analysis: *p* ≤ 0.01—highly significant; 0.01 < *p* ≤ 0.05—significant; *p* > 0.05—not significant. The means denoted by the different letters (A, B) differ statistically significantly (*p* ≤ 0.05).

**Table 3:** The effect of sex (gilts vs. barrows) on the levels of the selected biochemical blood parameters in pigs.

Biochemical Blood Parameters	Sex						SE	*p*
	G			B				
	*n*	$\bar{x}$	SD	*n*	$\bar{x}$	SD		
ALP (U/L)	60	132.60	34.91	60	132.61	38.01	4.89	0.873
ALAT (U/L)	60	58.92	12.09	60	56.50	14.26	1.74	0.411
ASPAT (U/L)	60	45.25	53.16	60	30.78	9.20	6.00	0.540
AMY (U/L)	60	2808.58	651.84	60	2609.28	644.44	88.48	0.443
GGTP (U/L)	60	37.56 ^B^	11.04	60	30.00 ^A^	10.10	1.53	0.022
Total protein (g/dL)	60	6.25	0.38	60	6.34	0.54	0.06	0.194
Albumin (g/dL)	60	4.47 ^B^	0.48	60	4.21 ^A^	0.32	0.06	0.021
Creatinine (mg/dL)	60	5.15	2.28	60	1.35	0.14	2.53	0.645
Urea (mg/dL)	60	24.25	7.39	60	26.96	11.61	1.22	0.154
Glucose (mg/dL)	60	83.56	11.41	60	82.89	8.53	1.42	0.788
Ca (mg/dL)	60	9.93	0.40	60	9.87	0.36	0.05	0.528
Mg (mg/dL)	60	2.30	0.17	60	2.34	0.21	0.03	0.665
Na (mmol/L)	60	142.56	2.60	60	143.56	2.07	0.33	0.054
Chlorides (mmol/L)	60	102.50	2.69	60	103.32	2.47	0.36	0.261
Inorganic phosphates (mg/dL)	60	7.94	0.54	60	7.81	0.76	0.08	0.307
Total cholesterol (mg/dL)	60	87.03	9.84	60	84.67	9.45	1.32	0.431
Triglycerides (mg/dL)	60	34.28	13.03	60	31.50	13.41	1.78	0.293
HDL cholesterol (mg/dL)	60	40.78	4.86	60	39.89	4.06	0.62	0.535
LDL cholesterol (mg/dL)	60	39.58	5.93	60	38.44	6.90	0.85	0.626
HDL/CHOL	60	46.94	3.41	60	47.50	5.40	0.56	0.762
Non-HDL cholesterol (mg/dL)	60	46.37	6.61	60	44.82	8.47	0.99	0.511
Cortisol (nmol/L)	60	173.54	72.68	60	168.15	69.09	9.65	0.858
β-endorphins (pg/mL)	60	897.50	320.37	60	1052.84	448.29	50.50	0.134

ALAT—alanine aminotransferase; ALP—alkaline phosphatase; ASPAT—aspartate aminotransferase; AMY—alpha-amylase; GGTP—gamma-glutamyl transpeptidase. Significance of the factor under analysis: *p* ≤ 0.01—highly significant; 0.01 < *p* ≤ 0.05—significant; *p* > 0.05—not significant. The means denoted by the different letters (A, B) differ statistically significantly (*p* ≤ 0.05).

## Data Availability

The original contributions presented in this study are included in the article. Further inquiries can be directed to the corresponding authors.

## Abbreviations

The following abbreviations are used in this manuscript:

ALAT	alanine aminotransferase
ALP	alkaline phosphatase
ASPAT	aspartate aminotransferase
AMY	α-amylase
GGTP	γ-glutamyl transpeptidase
